# Cure of intravascular NK/T-cell lymphoma of the central nervous system by allogeneic hematopoietic cell transplantation

**DOI:** 10.1038/s41409-022-01734-2

**Published:** 2022-06-09

**Authors:** Julia Meissner, Michael Schmitt, Mindaugas Andrulis, Leonille Schweizer, Sascha Dietrich, Bettina Alber, Inga Harting, Felix T. Kurz, Uwe M. Martens, Anthony D. Ho, Carsten Müller-Tidow, Peter Dreger

**Affiliations:** 1 Department of Medicine V, University of Heidelberg, Heidelberg, Germany; 2 Department of General Pathology, Institute of Pathology, University of Heidelberg, Heidelberg, Germany; 3 Department of Neuropathology, Institute of Pathology, University of Heidelberg, Heidelberg, Germany; 4 Department of Neuroradiology, University of Heidelberg, Heidelberg, Germany; 5 Department of Radiology, German Cancer Research Center, Heidelberg, Germany; 6 Department of Hematology/Oncology, Clinics Heilbronn GmbH, Heilbronn, Germany; 7 Present Address: Institute of Pathology, Klinikum Ludwigshafen, Ludwigshafen, Germany; 8 Present Address: Institute of Neuropathology, Charité, Berlin, Germany

**Keywords:** T-cell lymphoma, Stem-cell therapies

## To the Editor:

Intravascular lymphoma is a rare aggressive lymphoma characterized by the selective growth of malignant cells of lymphoid origin within the lumina of small vessels. On histological and immunohistochemical examination, most reported cases meet the criteria of intravascular large B-cell lymphoma [1] and have been categorized as a distinct entity in the current WHO classification [2]. Although mentioned as rare variants, intravascular lymphomas of NK/T-cell derivation (around 2% of reported cases) have not been defined as a separate entity. Since the initial description of a case of intravascular NK/T-cell lymphoma (IVNKTL) in 2003 [3], about 30 patients, predominantly with involvement of skin and/or central nervous system (CNS), have been reported (recently reviewed by Zanelli et al. [4]). Treatment strategies in patients with IVNKTL are not well defined, and especially in patients with disease not confined to the skin, the prognosis is extremely poor with no long-term survivor reported to date [4].

**Fig. 1:**
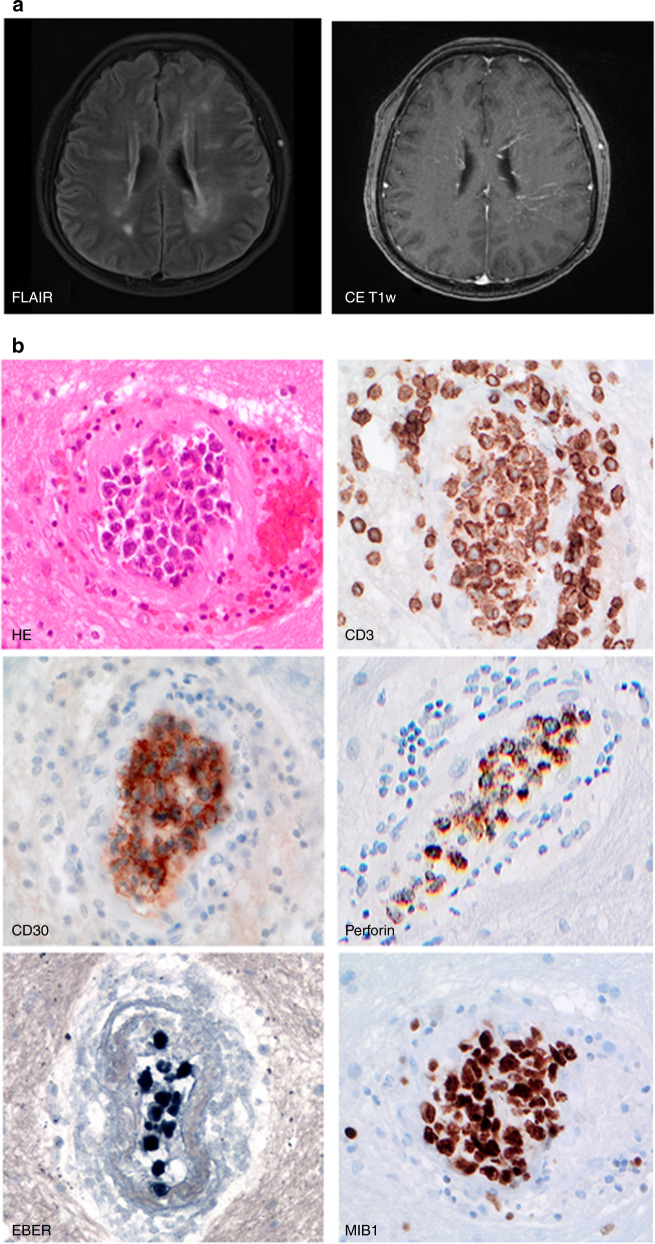
Magnetic resonance images of the brain and morphology and immunophenotype of intravascular NK/T-cell lymphoma at first presentation. **a** Axial fluid-attenuated inversion recovery (FLAIR) imaging showed multiple hyperintense spots in the bilateral watershed area as well as band-like periventricular white-matter hyperintensity. Contrast-enhanced T1-weighted (CE T1w) imaging revealed increased (peri-)vascular contrast enhancement, consistent with radiating, transmedullary vessels more pronounced in the left hemisphere. **b** Histological sections of the patient’s brain tissue (×400 original magnification) reveal large intravascular blasts in the lumina of vessels (H&E, hematoxylin eosin staining). The tumor cells show cytoplasmic expression of CD3, positivity for CD30, perforin, and EBER as well as a high proliferation rate as detected by Ki67/MIB-1 labeling.

Here, we describe a case of long-term remission after allogeneic hematopoietic cell transplantation (alloHCT) in a patient with IVNKTL with isolated CNS involvement. A 53-year-old male patient was admitted with progressive aphasia, gait abnormalities, cognitive impairment, and altered personality in October 2013. Examination of cerebrospinal fluid revealed Epstein-Barr virus (EBV, virus load 47,400 genome equivalents per ml). Cranial magnetic resonance imaging (MRI) displayed vascular and perivascular contrast enhancement as well as predominantly left-hemispheric, patchy, and partially confluent, T2-hyperintense white-matter abnormalities (Fig. 1a). MRI of the spine and computed tomography of the chest and abdomen showed no abnormalities. A random left frontal biopsy revealed brain tissue with large to medium-sized blastic cells with irregular nuclei and abundant pale cytoplasm that were exclusively lodged in the lumina of small vessels (Fig. 1b). Immunohistochemical staining showed a cytotoxic T-cell-like phenotype of the neoplastic cells with positivity for cytoplasmic CD3, CD56, perforin, and granzyme B as well as high cellular proliferation as measured by Ki67 (90%). The atypical cells were positive for EBV by in situ hybridization for EBV-encoded small nuclear RNA (EBER). PD-L1 and TCR expression were not examined. Around tumor-involved vessels, a sparse inflammatory infiltrate composed of small T lymphocytes was observed. T-cell receptor clonality and further conventional and molecular cytogenetic profiling could not be obtained due to limited material. Neither in cerebrospinal fluid nor in peripheral blood or bone marrow a clonal lymphoid population could be detected by flow cytometry.

Thus, diagnosis of IVNKTL with isolated CNS involvement was established, and the patient was treated aggressively with two courses of SMILE (methotrexate 2 g/m^2^ day 1, ifosfamide 1.5 g/m^2^ days 2, 3, 4, dexamethasone 40 mg days 2, 3, 4, etoposide 100 mg/m^2^ days 2, 3, 4, asparaginase 6000 IE/m^2^ days 8, 10, 12, 14, 16, 18, and 20). Brain MRI follow-up revealed partial regression of the perivascular contrast enhancement as well as of the confluent white-matter changes, whereas the neurological symptoms remained unchanged. Treatment was switched to a high-dose cytarabine-based regimen (cytarabine 2 × 2 g/m^2^ days 1–2, mitoxantrone 10 mg/m^2^ days 2–3; HAM). Given the high-risk nature of non-nasal NK/T-cell lymphoma [5] and in view of the refractory clinical course, subsequent alloHCT was planned. Treatment with altogether four courses of HAM led to significant clinical improvement with only residual abnormalities on brain MRI. EBV load was durably cleared. In June 2014, the patient underwent alloHCT from his HLA-identical, EBV-seropositive sister after conditioning with fludarabine and 6 gray (Gy) fractionated total body irradiation (TBI). Graft-versus-host disease (GVHD) prophylaxis consisted of cyclosporine and mycophenolate mofetil. Within the first 100 days after alloHCT, the patient developed grade 2 acute GVHD of skin and gut that was steroid-responsive. Peripheral blood at 3 months post-alloHCT showed full donor chimerism. Immunosuppression could be permanently discontinued on day +245 (February 2015). In October 2017, histologically confirmed mild chronic liver GVHD developed and required intermittent treatment with systemic steroids. However, the patient remained progression-free and is alive off systemic immunosuppression almost 8 years after transplantation with full donor chimerism and without clinical or imaging evidence for lymphoma recurrence.

To our knowledge, this is the first description of a long-term survivor of IVNKTL. Since it is highly unlikely that high-dose ara-c/mitoxantrone salvage could have caused sustained disease eradication in IVNKTL primary refractory to a regimen as aggressive as SMILE, the curative treatment effect observed here needs to be attributed to the consolidating alloHCT. It is well documented that alloHCT can provide durable disease control in patients with relapsed/refractory peripheral T-cell lymphoma including those with NK/T-cell lymphoma [6–8]. Evidence for the notion that graft-versus-lymphoma activity contributes to alloHCT efficacy in NK/T-cell lymphoma is based on the observation of plateaus in the relapse incidence curves even after reduced intensity conditioning, and the anecdotal success of donor lymphocyte infusions [6]. A graft-versus-lymphoma effect would be also in keeping with the GVHD episodes observed in our patient.

Since NK/T-cell lymphomas are known to be sensitive to radiotherapy, one might speculate about the potential role of TBI in treatment success. However, to date, there is no sound evidence that TBI-based conditioning might be superior to chemotherapy conditioning in NK/T-cell lymphoma although the data basis for this is clearly limited [6]. Moreover, our patient received only reduced-dose TBI because he had been considered to be incapable of withstanding full-intensity myeloablative conditioning.

Finally, IVNKTL was associated with EBV which is a potent T-cell immunogen. EBV-specific T lymphocytes have been reported to exert anti-tumoral and anti-leukemic effects [9] and thus may have played a role in IVNKTL clearance observed here.

Regarding IVNKTL not confined to the skin, we are aware of only a single case of alloHCT for this entity. This was a young woman who underwent haplo-identical transplantation for the refractory disease after having failed four lines of intensive chemotherapy [10]. Although transplant details including conditioning and disease response were not reported, the fact that this patient survived nine months before she succumbed to acute GVHD may suggest that IVNKTL progression at least was not a predominant contributor to the fatal outcome. If we consider, nevertheless, our patient’s course as proof of principle for the effectiveness of consolidating alloHCT in IVNKTL, the optimum timing of transplantation needs to be discussed. A recent randomized trial failed to prove the superiority of consolidating first-line alloHCT over autologous hematopoietic cell transplantation in patients with peripheral T-cell lymphoma [11]. However, this trial did not include patients with NK/T-cell lymphoma. Thus, given the poor efficacy of standard first-line therapy in IVNKTL [4], there might be some rationale for considering alloHCT as soon as a response is achieved, similar to other highly aggressive rare peripheral T-cell lymphoma [12].

In summary, our report suggests that long-term survival can be achieved in patients with non-cutaneous IVNKTL and provides evidence that alloHCT should be proactively considered in patients with the chemosensitive disease.

## Data Availability

For additional information, please contact the corresponding author.
